# Lebanon’s Native Oenological *Saccharomyces cerevisiae* Flora: Assessment of Different Aspects of Genetic Diversity and Evaluation of Winemaking Potential

**DOI:** 10.3390/jof7080678

**Published:** 2021-08-23

**Authors:** Marie-José Ayoub, Jean-Luc Legras, Pierre Abi-Nakhoul, Huu-Vang Nguyen, Rachad Saliba, Claude Gaillardin

**Affiliations:** 1 Department of Food Sciences and Technologies, Faculty of Agricultural and Veterinary Sciences, Lebanese University, Beirut 14-6573, Lebanon; pabinakhoul@ul.edu.lb (P.A.-N.); rachadsaliba@hotmail.com (R.S.); 2 SPO, University of Montpellier, INRAE, Institut Agro, F-34060 Montpellier, France; jean-luc.legras@inrae.fr; 3 CIRM-Levures, SPO, University of Montpellier, INRAE, Institut Agro, F-34060 Montpellier, France; vangng08@yahoo.fr; 4 AgroParisTech, Micalis UMR 1319, CBAI, F-78850 Thiverval-Grignon, France; c_gaillardin@orange.fr; 5 INRA, Micalis UMR 1319, CBAI, F-78850 Thiverval-Grignon, France

**Keywords:** *Saccharomyces cerevisiae*, biodiversity, natural fermentation, indigenous yeast, wine aroma

## Abstract

A total of 296 isolates of *Saccharomyces cerevisiae* sampled from naturally fermenting grape musts from various locations in Lebanon were typed by interdelta fingerprinting. Of these, 88 isolates were compared with oenological strains originating from various countries, using microsatellite characterization at six polymorphic loci. These approaches evidenced a large diversity of the natural oenological Lebanese flora over the territory as well as in individual spontaneous fermentations. Several cases of dominance and perenniality of isolates were observed in the same wineries, where fermentations appeared to involve lineages of sibling isolates. Our work thus evidenced a “winery effect” on strains’ relatedness. Similarly, related or identical strains were also detected in vicinal wineries, suggesting strain circulation within small geographical areas and a further “vicinity effect”. Moreover, and despite its diversity, the Lebanese flora seemed interrelated, on the basis of microsatellite loci analysis, in comparison to worldwide communities. We finally tested the ability of 21 indigenous strains to act as potential starters for winemaking. Seven of them passed our pre-selection scheme and two of them at least may be good candidates for use provided pilot-scale assays confirm their suitability.

## 1. Introduction

“They shall blossom like the vine; their fame shall be like the wine of Lebanon” [1]. “Wine, sweet and abundant, select wine…the choice wine of Lebanon” [2]. These are only a few of many testimonies praising the renown and evoking the oldness of wine production in Lebanon, while modern archaeological and scientific evidence have backed up ancient literature. The discovery of shipwrecks from the 8th century BC carrying Phoenician wines from Tyr [3] and the recent unearthing of an iron-age plaster from a Lebanese wine press [4] date the production of Lebanese wines back to the Phoenician era at least. On another level, genetic studies have suggested that the Lebanese oenological *Saccharomyces cerevisiae* flora seems to be ancestral to other worldwide oenological floras [5]. These findings are in line with the assumption according to which Phoenicians have played a key role in the introduction of grapevine culture and wine consumption to Europe and other parts of the world [6]. In parallel, wine yeasts have been dispersed through co-migration with grapes and wine following human migration routes [5,7,8]. This ancient tradition of winemaking has been perpetuated to this day in Lebanon and natural fermentations are still carried out as they were in ancient times.

*Saccharomyces cerevisiae* is known to be the major contributor to leading and finishing such natural wine fermentations, as evidenced by the many studies devoted to the identification and dynamics of the natural floras of fermenting grape musts [9,10,11,12,13], and its presence has been confirmed in one of the most ancient known wine lees [14]. It has been reported that many *S. cerevisiae* strains usually coexist in natural fermentations [15,16,17,18,19,20,21,22,23]. Such complexity is observed even when musts are inoculated with selected starters, though natural equilibrium is affected in such cases [24,25,26]. Several studies have indicated that most of this biodiversity is represented by small, subdominant populations whereas a few strains dominate the fermentation or phases of it [19,27,28,29,30]. Moreover, in a particular winery or wine producing area, some strains seem to maintain themselves over several years [28,29,30,31,32] and disseminate around these places [33,34,35]. These observations coupled with the more recent reporting on the existence of a regional effect on genetic differentiation of microbial communities [36,37,38,39,40], and *S. cerevisiae* populations in particular [41,42,43,44], have brought forth a theory about the existence of “terroir” specific populations or strains that may be representative of their localities. Along with the observations of regional genetic differentiation, evidence is piling up about an associated phenotypic differentiation in the resulting wines in terms, for example, of volatile composition [45,46]. These regional genetic and phenotypic signatures of *S. cerevisiae* communities may suggest local adaptations and it may be supposed that local strains may have been selected by or have co-evolved with specific wine making conditions. Autochthonous strains may thus be particularly desirable for local wine productions. In this regard, a growing number of studies is evaluating the potential of using indigenous strains in wine productions [47,48,49,50], which is supposed to overcome the potential standardization and loss of typicality introduced by the use of the same commercial active dry yeasts worldwide [12], and therefore improve the sensory characteristics of wines by producing typical and distinguished aromas linked to the terroir or to specific wine types [48,49,50,51,52].

Most *S. cerevisiae* ecological studies have concerned European countries like France [19,22], Spain [21,29], Italy [53,54], Portugal [33] or Greece [17], and the majority of them studied the vineyard fermenting flora, which does not necessarily represent the natural fermentations occurring in wineries. However, to our knowledge, no extensive biogeographical diversity study has concerned the *S. cerevisiae* oenological flora in the Middle Eastern region, one of the cradles of winemaking, even though a recent study addressed *S. cerevisiae* diversity in a Lebanese winery [55]. It thus seemed interesting to explore different aspects of the fermenting *S. cerevisiae* flora’s diversity in Lebanon. A large-scale biodiversity study was consequently conducted over the whole Lebanese territory for the first time, in order to explore the diversity and the geographical distribution of the indigenous oenological *S. cerevisiae* flora and to examine the possible existence of a locality or wine type effect. Since industrial winemaking in Lebanon is a relatively recent activity in comparison to its long history in this field, and since it is mainly confined to a limited geographical area, most of its natural flora should have been preserved in traditional wineries from genetic exchange with non-Lebanese strains. To assess whether the Lebanese flora possesses any specificity, we compared isolates from this indigenous population to communities originating from different countries. We based our various analysis on interdelta fingerprinting [56] and microsatellites typing [57], two easy-to-implement approaches that have proved to be efficient in differentiating or inferring relatedness of *S. cerevisiae* strains. In a complementary approach, and assuming a role for *S. cerevisiae* strains in the development of particular and characteristic wine traits, identification of suitable strains from Lebanon may be relevant for the future development of winemaking in this country. The strain’s choice is therefore essential, and we tested in this study the ability of 21 indigenous strains to conduct wine fermentations to later act as potential starters for local winemakers.

## 2. Materials and Methods

### 2.1. Isolation and Analysis Scheme

A total of 149 samples of naturally fermenting grape musts were collected over four consecutive years (I, II, III, IV) from sweet or dry (red, white or rosé) wines produced according to classical methods of fermentation.

Different aspects of Lebanese *S. cerevisiae* flora’s inter-diversity were studied using interdelta region amplification. These are summarized in Table 1. The first aspect aimed at obtaining a large geographical survey of *S. cerevisiae* strains in Lebanon. To reach this goal, 79 wineries scattered among 51 villages (Figure S1) spread all over the country and representing all regions producing naturally fermented wines were sampled, mainly during year III, and a single winery was represented by 1 to 14 *S. cerevisiae* isolates. The second aspect to be studied was diversity estimation within wineries. For this purpose, 9 to 10 isolates were studied per vat from 12 vats originating in 9 wineries (Table 1 and Table S1). The third matter to be addressed was the study of perenniality in wineries. To achieve this, five wineries were followed over two years, one over three years, and one over four years (Table 1 and Table S1). Each of these 7 wineries was represented during analysis in a given year by 4 to 14 isolates (from the same or different vats) while numbers below 4, even if available for a given year, were not considered for analysis.

At another level, and to assess the specificity, if any, of the Lebanese strains, they were compared to non-Lebanese isolates. This issue was addressed using microsatellite typing that was used to compare 88 Lebanese strains with non-Lebanese strains. These included strain S288C as well as 11 commercial wine strains (Table S2): Levuline BRG, Levuline CER, Levuline CHP, Ceres C2C, Levuline C19, EG8, Levuline FB, Levuline Killer, Montbazin 1 (in this paper MBZ), Levuline Primeur, Montrachet Davis 522 (in this paper 522D), plus a subset of 237 *S. cerevisiae* oenological strains characterised by Legras et al. [5] originating from different villages and regions in various countries (Table S3). The same previous eleven commercial strains and eight non-oenological strains were analysed with interdelta fingerprinting. The non-oenological strains included five strains isolated on various substrates (CLIB 409, CLIB 412, CLIB 413, CLIB 414, CLIB 415 and CBS 1171NT) as well as strains S288C and CBS 1907 (Table S2).

### 2.2. Strains Identification

Single colonies were isolated from cultures of appropriate dilutions of the collected musts, grown on YPD solid medium (yeast extract 10 g·L^−1^, peptone 10 g·L^−1^, D-glucose 10 g·L^−1^, agar 20 g·L^−1^), and incubated at 28 °C. A preliminary identification was performed using biochemical tests [58] and API ID 32C strips (Biomérieux, Craponne, France). PCR/RFLP of ribosomal NTS2 region was used [59] in order to confirm affiliations to *S. cerevisiae*. Strains were stored at −80 °C in YPD liquid medium supplemented with 25% glycerol.

### 2.3. Inter-Delta Fingerprinting

A standard procedure was used for DNA extraction [60]. Primers used for amplification were delta 12 (5′-TCAACAATGGAATCCCAAC-3′) and delta 21 (5′-CATCTTAACACCGTATATGA-3′) [56]. Amplification reactions were performed in 25 μL mixtures containing Ex-Taq buffer (2 mM Mg^2+^), Ex-Taq (0.75 U-Takara), 200 μM of each dNTP, 0.5 μM of each oligonucleotide primer and 10–50 ng yeast DNA. A 9600 Perkin-Elmer Cetus thermal cycler was used, and the following program applied: initial denaturation for 3 min at 95 °C, then 35 amplification cycles (95 °C for 30 s, 45 °C for 40 s, 72 °C for 90 s), and a final extension step of 5 min at 72 °C. The amplification products were separated on 0.8% agarose gels in 0.5× TBE buffer. Gels were stained with ethidium bromide, visualized and photographed under UV light. Gel pictures were normalized using the Bionumerics program (Applied Maths, version 1.01) and the profiles were clustered using the neighbour-joining aggregation method based on Dice coefficient from the same software.

### 2.4. Microsatellites Analysis

Six highly resolutive microsatellite loci previously tested for their suitability in *S. cerevisiae* typing were used [57]. The loci were SCYOR267C, YPL009c, C4, C5, C10 and C11. PCR reactions were performed in 20 μL of Qiagen multiplex PCR kit mixture containing 10–50 ng of template DNA. Primers were used at the concentrations of 0.1 μM except those of locus C4, the concentrations of which were of 0.2 μM. The reverse primers were labelled with the fluorescent dyes VIC, 6-carboxyfluorescein (6-FAM), benzofluorotrichlorocarboxy-fluorescein (NED) (Applied Biosystems, Cheshire, UK). The first dye was used for loci C5, C10 and SCYOR267C, the second for YPL009c and C11 and the third for C4. Amplifications were performed according to the following program: 15 min at 95 °C, 34 cycles (30 s at 95 °C, 2 min at 57 °C and 1 min at 72 °C), and a final cycle of 10min at 72 °C before holding at 4 °C. Electrophoresis on 2% agarose gels was performed to check for successful amplifications. Microsatellites raw data were analysed using The Genescan software (Applied Biosystems, version 3.7). The different alleles corresponding to each isolate were computed to generate a similarity matrix (according to Dice coefficient), which was used to generate an aggregation of the isolates by the PHYLIP software (version 3.6) [61] according to the neighbour-joining method.

### 2.5. Strains Choice for Technological Characterization and Fermentations

Twenty-one indigenous *S. cerevisiae* strains (Table S4) originating from different locations and wine types and the commercial oenological strain 522D were studied. The 22 yeast strains were assessed for their ethanol resistance and their H_2_S producing abilities. For ethanol resistance tests, yeasts pre-cultures were used to inoculate liquid YPD media containing 0, 12, 14 and 16% (*v*/*v*) ethanol that were incubated at 28 °C. These in turn were used to inoculate solid YPD plates after 48 h. Viability (in%) at the different ethanol concentrations was determined as being the ratio between the number of colonies obtained from liquid cultures supplemented with ethanol and the number of colonies from ethanol free cultures. For H_2_S production tests, the strains were grown on Biggy Agar medium (Bismuth Sulphite Glucose Glycine Yeast, Difco, Sparks, MD, USA) previously evaluated for its suitability to predict strains behaviour in real fermentations [62] and recommended by OIV [63]. The production of H_2_S results in a variety of colony colourations. White to creamy colonies were considered to correspond to no/low levels of H_2_S, light brown to brown colonies to medium levels, and dark brown to black colonies to high levels of H_2_S.

### 2.6. Lab-Scale Fermentations and Chemical Analysis of Wines

Fermentations were performed at 28 °C in 1 L flasks containing 800 mL of grape must (pH 3.4; 200 g/L sugars; 247 mg/L assimilable nitrogen) composed of cabernet sauvignon (50%), cabernet franc (25%) and merlot (25%). They were carried out in duplicate for each strain. Their evolution was followed daily by the measure of Brix degree (corrected for ethanol presence according to Weast [64]) and cell concentrations (UFC/mL). Brix stabilization for three days indicated the end of fermentations. The experimental wines produced were centrifuged (4000× *g*, 5 min), filtered and conserved at −80 °C until analysis. Wines were analysed for residual sugars, ethanol, glycerol, acetaldehyde, ethyl acetate and some major wine higher alcohols (n-propanol, isobutanol and isoamylic alcohol) concentrations. Sugars concentrations were determined by high performance liquid chromatography (HPLC) using a Supelcosil LC-NH2 column (58338, Sigma-Aldrich, Darmstadt, Germany) and a refractive index (RI) detector. The mobile phase consisted of an 80% (*v/v*) acetonitrile/water solution (Romil, Cambridge, UK). The flow rate was 1.5 mL/min and the injection volume 20 μL. Glycerol concentration was determined by an enzymatic kit according to manufacturer’s instructions (K-GCROL) (Megazyme International, Wicklow, Ireland). Ethanol and aromatic compounds (acetaldehyde, ethyl acetate, n-propanol, isobutanol and isoamyl alcohol) were analysed by gas chromatography (GC) using an 80/120 Carbopack B AW/5% Carbowax 20 M packed column (11812, Sigma-Aldrich, Darmstadt, Germany) and a flame ionization (FID) detector with nitrogen as the carrier gas (18 mL/min flow rate). Injection of 1.5 μL was carried out at a temperature of 170 °C while the FID detector temperature was 200 °C. For aromatic compounds, a column temperature gradient of 70 °C to 170 °C at 7 °C/min was used, while the temperature for ethanol was 90 °C.

### 2.7. Fermentation Results Analysis and Sensory Paired Comparison Test

For technological and fermentation data, the Xlstat software (Addinsoft, version 1.1.1084) was used to perform descriptive statistics, analysis of variance, establish correlations (according to Pearson coefficient) and carry out principal component analysis (PCA). The experimental wines of two strains selected after results analysis were subjected to a paired comparison test by a wine expert who evaluated the following wine characters intensities: aromatic, undesirable flavours, tannins and body.

## 3. Results

### 3.1. Diversity of the Natural Lebanese Oenological Flora across the Territory

To explore the biodiversity of *Saccharomyces cerevisiae* across the Lebanese territory, a total of 296 Lebanese isolates confirmed as belonging to *S. cerevisiae* and having produced exploitable interdelta patterns were analysed. Our amplification conditions yielded a total of 31 bands in the population’s profiles, most of which were found at frequencies < 20% of the isolates population while only 5 were found at frequencies > 50%.

Among the 296 Lebanese isolates analysed, 226 different interdelta patterns were recorded revealing a high genetic diversity (76.35% different genotypes) within the Lebanese population. These figures are very close to those of other studies of *S. cerevisiae* populations like Börlin et al. [19] or Gayevskiy and Goddard [41], who reported, respectively, 77.02% different genotypes from Sauternes appellation of French Bordeaux region, and 77.62% from New Zealand, both by microsatellites analysis. Within the Lebanese population, 182 patterns (80.53% of the total number of different patterns) belonged to a unique isolate each (all of which representing 61.48% of the total isolates population). A total of 37 patterns were each shared by 2 to 10 isolates originating from the same winery, mainly from the same fermenting vats and less frequently from different vats (the same year or through different years), while seven profiles were each shared by two isolates from different wineries. All patterns from different wineries were thus different in pairwise comparisons at the exception of these seven cases of patterns identities. All the cases of shared profiles will be discussed later in more detail. Excluding 100% identities, pairwise similarities between profiles ranged from 0 to 96.3% according to the binary Dice coefficient based upon the presence of common bands.

**Figure 1:**
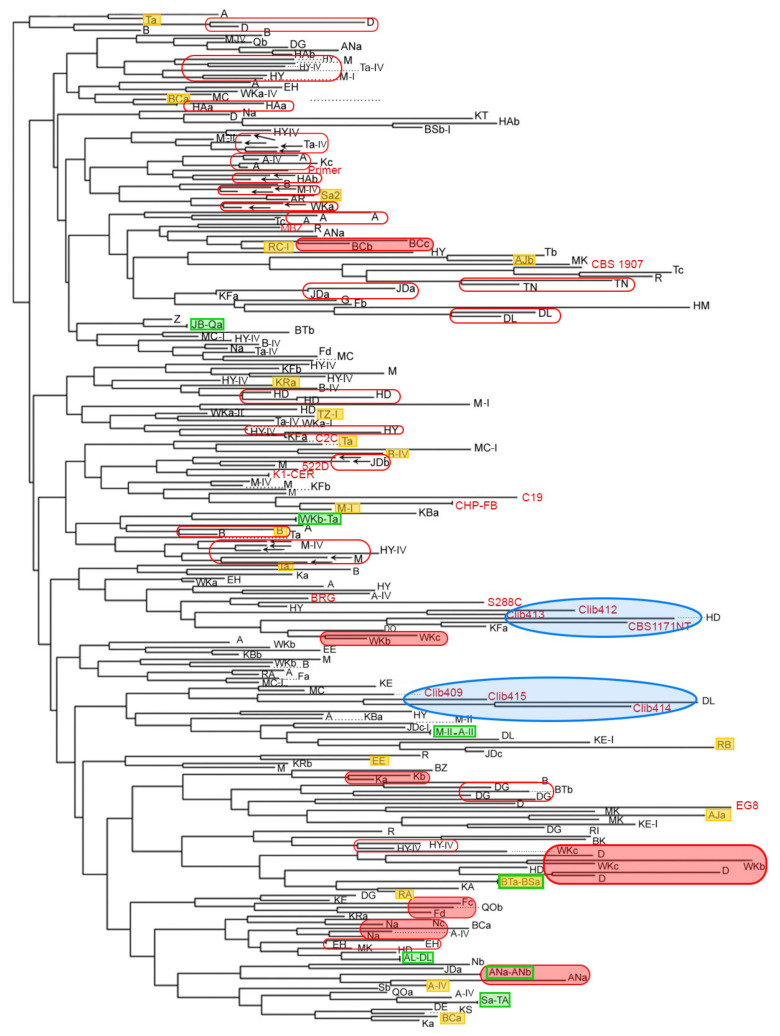
Clustering of 233 Lebanese and 19 non-Lebanese isolates according to interdelta fingerprinting. Lebanese isolates are indicated by the initials of their corresponding wineries. Redundant isolates from the same vats or wineries are not represented. Wineries names are indicated by capital letters representing the initials of the village (e.g., HA). When several wineries are examined per village, uppercase letters are followed by lowercase letters (e.g., HAa, HAb). Most of the isolates were sampled in year III unless otherwise indicated by I, II or IV at the end of the isolate name. Non-Lebanese isolates are in red. Red non-filled and red-filled rectangles correspond respectively to highly similar isolates from the same winery, or from wineries of the same village. Yellow and green filled rectangles correspond respectively to isolates taken from sweet wines or identical isolates from different wineries. Blue ovals highlight two clusters of non-oenological isolates.

The clustering of 233 Lebanese isolates representing the 226 different patterns, and including all the isolates harbouring unique patterns, as well as one representative of each identical pattern originating from the same winery and both representatives of identical patterns in provenance of different wineries, is shown in Figure 1. No clear relation could be observed between strains clustering and geographical origin. However, some clustering of strains originating from the same or neighbouring wineries was observed (see below). When the wine type process was considered, no particular clustering of isolates was neither observed, and identical or very similar strains (90% and more) could indifferently be isolated from dry (all types) or sweet wines (indicated by yellow filled rectangles on Figure 1).

### 3.2. Diversity of the Oenological Flora in Fermenting Vats and Wineries

The frequent occurrence of identical patterns from the same vats or wineries described above led us to investigate in more details nine selected wineries. In each of these wineries, samples were taken from one or several vats.

**Figure 2:**
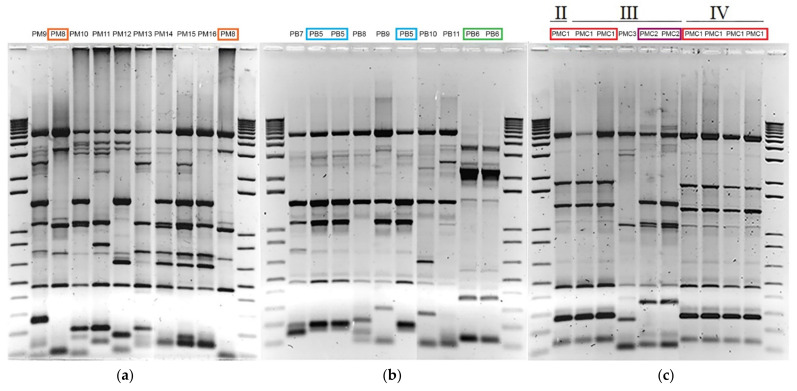
Diversity, dominance and perenniality of *S. cerevisiae* isolates in wineries. (**a**) A single dominant profile (PM8, framed in orange) is found in the same vat of winery M. (**b**) Two dominant profiles (PB5 in blue and PB6 in green) are found in the same vat of winery B. (**c**) One profile (PMC1 in red) of winery MC is found during several years and in different vats of year III. PMC2 (in violet) is found in the same vat of year III.

During years III and IV, and in the cases where only a single vat was sampled per winery, 10 or 9 isolates were studied from one vat (while numbers below nine were not considered during analysis, like for winery WKa/year III, which was not taken into account due to only having four isolates—Table S1). A diversity of profiles was observed between isolates of the same vat (where the number of different patterns varied between 0 and 100%, with a mean of 76.33% different patterns per vat) but similar patterns were nonetheless observed repeatedly (Figure 2a,b). One or two patterns were found more frequently than the others in 11 out of 12 such vats analysed (Table S1). These “dominant” isolates represented at least 20 or 30% of the sampled population of a single vat. Three of them represented 40 to 50%, and two, 80 and 100% of the sample (Table S1). Though these proportions (and those that will follow) have to be taken with caution, since the sampled population per vat/winery was small, they nonetheless give an idea about the existence of frequent or stable strains. We will therefore refer to the frequent strains as “dominant” for convenience.

During years I, II and III, and in six out of eight cases in which several (two or more) vats were sampled, similar patterns were repeatedly observed between the different vats of the same winery (Table S1). The proportion of these patterns varied between 14 and 57% of the winery population (not taking into account winery T, year II, where only two isolates were available). For instance, in winery A (year III) two patterns (PA4 and PA11) were found in two vats each, representing individually 14% of the sampled population from six vats. In winery MC and year III, though the total number of isolates was lower, one pattern (PMC1), dominant from a single vat of year IV, was detected in two out of 3 vats and represented 57% of the sampled population from the three vats (Table S1 and Figure 2c).

### 3.3. Stability of Strains in Wineries

We looked in seven of the above wineries at the possible perenniality of strains. Winery M was followed throughout all four years, while winery MC was followed for three years with an additional available isolate from year II. Five other wineries were followed through years III and IV, with 4 to 14 isolates per winery. Three of these also had available isolates for one or two years (Table S1). In five of the seven wineries, the same patterns were found for more than one year (Table S1). In two wineries (WKa and MC), one pattern was found for two or three consecutive years. In both cases this pattern was dominant over two years, representing in the first winery (WKa) 75% of the year III population and 80% in year IV, and in the second winery (MC), pattern PMC1 represented 57% of the year III population and 100% in year IV. PMC1 had been already detected in year II, where only a single strain had been tested (Figure 2c). Non-dominant patterns were also found for several years, like in winery HY, where two isolates were detected for two consecutive years, while in winery M, one pattern was found for two non-consecutive years (Table S1). In winery A, pattern PA4, mentioned earlier as being dominant, plus three other non-dominant patterns (PA3, PA5 and PA6) were isolated in years III and IV. It therefore seems that strains perenniality and persistence in wineries are not related to their dominance in a given fermentation or winery.

### 3.4. Winery and Geographic Vicinity Effects on Strain Relatedness

Global comparison of isolates evidenced repeatedly clusters of two or more isolates originating from the same wineries, either taken the same year or through different years (too many cases to be shown on Figure 1, so only a few are indicated by red rectangles), from different wineries of the same village (some are indicated by red filled rectangles on Figure 1) or from villages found within a range of 25 km. These strains were closely related, and many had pairwise similarity scores above 90%. As for 100% identities, most were of isolates in provenance of the same wineries while only seven cases were of isolates in provenance of different locations (green rectangles—Figure 1). Among these, one concerned wineries of the same village (ANa/ANb), three concerned wineries from vicinal villages found within a range of 25 km (BSa/BTa and A/M within 5 km each and AL/DL within 25 km), and three were of wineries located in more distant villages (JB/Q, Sa/TA, WKb/Ta).

**Figure 3:**
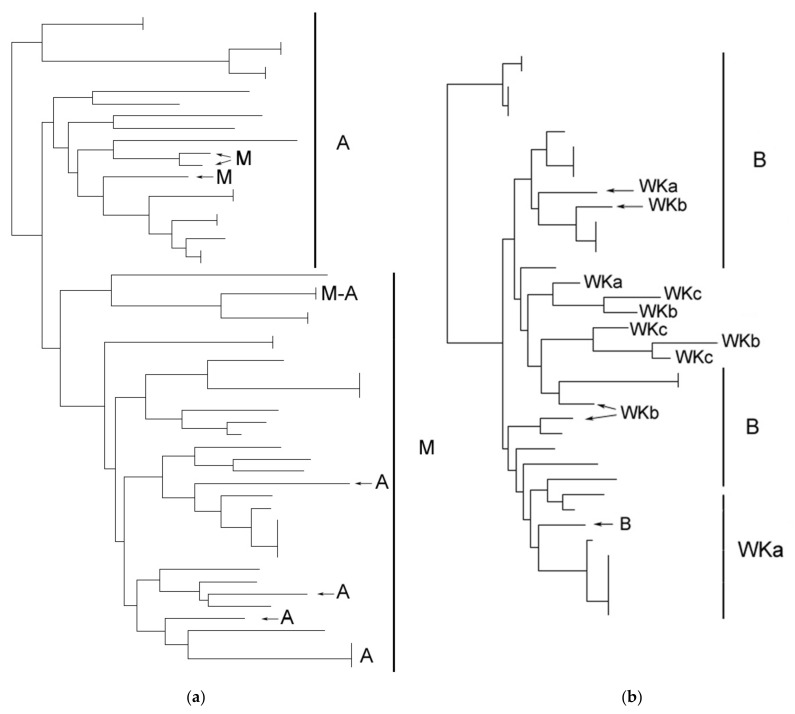
Interdelta clustering of isolates from geographically close wineries: (**a**) wineries A and M; (**b**) winery B and 3 wineries from village WK (WKa, WKb and WKc). Only the provenance of the isolates is indicated on the figure.

We thus wanted to verify whether strain relatedness from proximate locations would be linked to geographical vicinity. We therefore chose to examine two pairs of vicinal villages found within a close range of 5 km (pair 1: M and A, and pair 2: B and WK). A clustering of isolates from these pairs of wineries had been observed in the global comparison, while a common interdelta pattern was found between wineries A and M. All available isolates, regardless of the isolation year, were consequently analysed from a single winery in the villages A, M and B. For the village WK, and in addition to the isolates of winery WKa presented in Table S1, isolates from two additional wineries, WKb and WKc, were included in the comparison (Figure 3).

The isolates from wineries M and A (Figure 3a) were clustered according to their provenance, suggesting the existence of winery specific lineages. This observation, coupled with the previous findings of perenniality and circulation of strains within wineries, tend to suggest a “winery effect” on oenological strains relatedness. When comparing isolates from winery B and wineries of the WK village, we further observed that the clustering was not only related to wineries but also to the village, since a grouping of isolates from different wineries of WK was observed. These results, added to the existence of an identical isolate in both A and M, show that in vicinal geographical locations extending over at least 5 km, wine fermentation may be conducted by a set of circulating related strains, suggesting a further “vicinity effect” on strains relatedness.

### 3.5. Comparison of Lebanese Isolates with Floras of Diverse Origins

We next wanted to consider the possible existence of a specific Lebanese *S. cerevisiae* community, which would be genetically distinguishable from strains isolated in other parts of the world. Clustering of the interdelta fingerprints did not evidence such a specificity: eleven oenological strains isolated outside from Lebanon as well as S288C and CBS 1907 were scattered among the Lebanese isolates and did not cluster separately except for commercial strains that seem to be clones or derived from each other like 522D, K1 and CER or CHP, FB and C19. On the contrary, six non-oenological strains (CLIB series and CBS 1171NT), isolated on different non-oenological (mostly fermented) substrates, clustered in two separate groups, suggesting that they are more related to each other than to oenological strains (blue ovals in Figure 1). Nonetheless, the small number of non-Lebanese strains included in this comparison may have created a sampling bias which did not permit to fully address this question.

**Figure 4:**
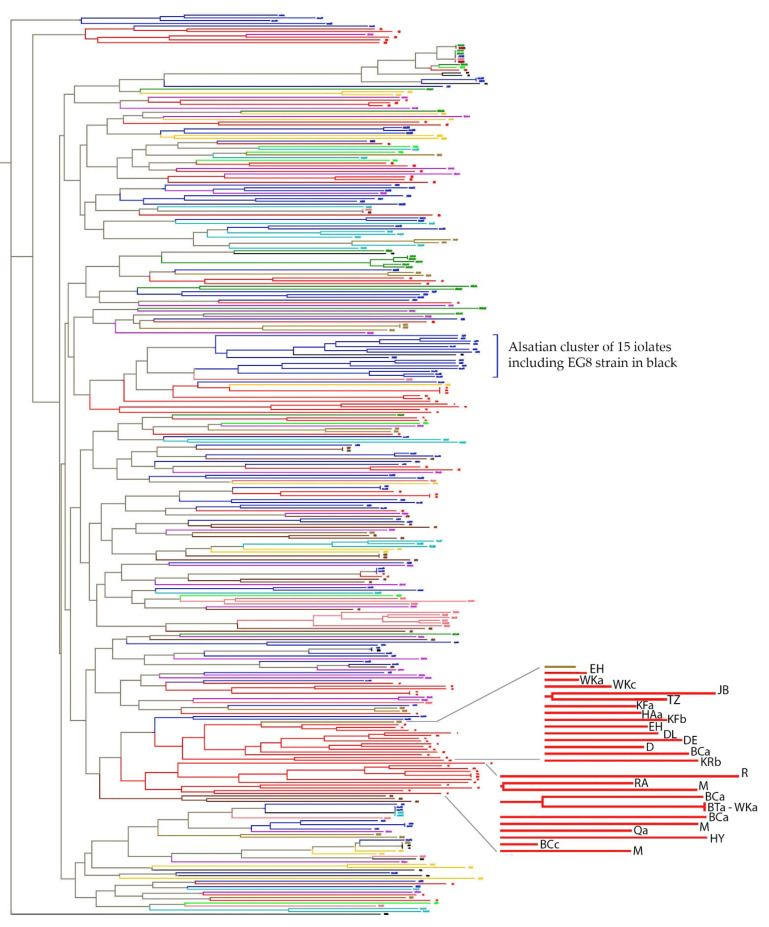
Clustering of Lebanese and worldwide oenological isolates according to microsatellites analysis. Lebanese isolates are in red. Isolates from Spain, South Africa, USA and Italy are respectively in violet, green, yellow and kaki. French isolates are coloured differently according to the region of isolation: those of Alsace, Montpellier, Côtes du Rhônes, Nantes, Cognac and Champagne are respectively in dark blue, light blue, brown, pink, light green and orange. Isolates in black are the 11 commercial strains and S288C. Enlargement of Lebanese strains shows their provenance.

To better comprehend this issue, we used a larger set of oenological strains and a different typing method. Microsatellite analysis of six polymorphic loci that had previously been shown to provide discriminatory and reproducible results while revealing relatedness of *S. cerevisiae* strains [57] was chosen to type 88 Lebanese isolates. These were selected so as to harbour different or identical interdelta patterns and to originate from different villages, different wineries of the same villages, and different vats or the same vats of single wineries. They were compared to 237 non-Lebanese wine strains originating from different villages and regions in different countries (Table S3), as well as to the same 11 commercial strains included in the interdelta comparison beside strain S288C. The clustering of all isolates, excluding eight Lebanese isolates that were identical to others from the same wineries, is shown in Figure 4.

Microsatellites typing confirmed most of the conclusions reached when using interdelta fingerprinting. Oenological floras from all regions and countries including Lebanon were found to be extremely diverse within and between the countries. In all countries or regions studied, the most frequent observed effect was the “winery effect”, as it was very common to find clusters of two or more strains originating from the same winery. Strains coming from different wineries of the same villages or limited geographical areas could also be found related (from Alsace or Nantes for example), though this grouping was not obvious for all the sampled regions and not systematic for all the isolates from a given region.

Surprisingly, however, a certain extent of clustering of Lebanese strains isolated from distant wineries was observed (Figure 4). This clustering was rather broader in terms of the number of isolates involved per cluster and of the geographical areas concerned than what was observed for other countries or regions, even when considering areas having a similar number of isolates as Lebanon (Alsace region or even France as a whole). Indeed, clustering of Lebanese strains was not related to the provenance of the isolates within the Lebanese territory, since the wineries involved were not necessarily vicinal and it involved villages scattered all over the territory. For example, strains from villages distributed from the extreme south (village R) to the extreme north (village Q) were involved in the same cluster (Figure S1 and Figure 4).

The only other observed large cluster was of 15 strains originating from Alsace. However, unlike the Lebanese isolates, these Alsatian strains all originated from a narrow geographical area, with nine of them being from the same winery (W4, Table S3), and they were all related to the commercial strain EG8, initially isolated from Alsace. These observations according to microsatellites typing suggest that the Lebanese flora, though diverse, is interrelated in comparison to worldwide floras.

### 3.6. Lab-Scale Fermentations and Technological Screening

Twenty-one indigenous strains originating from the same or different wineries, different geographic locations and all available wine types (Table S4) were chosen to conduct lab-scale fermentations. The commercial oenological strain 522D described in the literature as being suitable for the inoculation of all types of wines was also included [65]. We determined for all experimental wines aromatic molecules that could negatively impact aroma at high doses, i.e., the most abundant ester (ethyl acetate), aldehyde (acetaldehyde) and higher alcohols (isoamyl alcohol, isobutanol and n-propanol). We also determined the glycerol content, alcoholic degree, total acidity and residual sugars to examine the completion of the fermentations. Additionally, we assessed for all the strains their ability to produce hydrogen sulphide and their viabilities at 12, 14 and 16% ethanol. Data for all parameters are reported in Tables S5 and S6.

Our results showed that the Lebanese indigenous strains of *S. cerevisiae* differed by their oenological capacities. Indeed, variance analysis of the studied parameters showed that all of them varied significantly with the strain (at the threshold *p* = 0.05), except total acidity (Tables S5 and S6). However, we could neglect the variations of alcoholic degree and residual sugars since they were caused by the extreme results of strains S1 and S11 that did not complete the fermentations and consequently lead to low alcoholic degrees (7.86 and 9.23%, respectively) and high residual sugars (53.25 and 35.65 g/L, respectively). The other strains lead to experimental wines that can be considered as dry, designation reserved according to OIV [66] for wines containing less than 4 g/L of residual sugar or 9 g/L, provided that the total acidity (expressed as g/L of tartaric acid) is not more than 2 g/L lower than the sugar content.

Eight indigenous strains (S5, S6, S7, S8, S9, S10, S19 and S21) had the ability to produce hydrogen sulphide at medium or high levels (Table S5). This compound, which gives wine a rotten egg odour, is detrimental, since its sensory perception threshold can be very low (1.1–1.6 μg/L), while strains of *S. cerevisiae* produce it in the range of 0 to ~300 μg/L [67,68]. We thus considered the ability to produce medium or high levels of H_2_S to be a selection factor, and all producing strains were eliminated from further analysis. This, along with ruling out strains S1 and S11, which did not complete fermentation, left 12 possible candidate strains for selection.

### 3.7. PCA Distribution of the Strains According to Oenological Parameters

**Figure 5:**
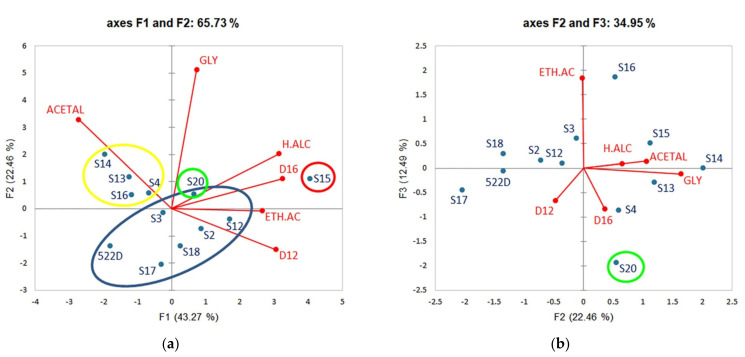
Principal component analysis (PCA) plots based on oenological parameters determined for 11 indigenous *S. cerevisiae* strains and 522D and their experimental wines. (**a**) Biplot of axis F1 and F2 explaining 65.73% of the variation. (**b**) Biplot of axis F2 and F3 explaining 34.95% of the variation. Variables: oenological parameters Observations: strains. ACETAL: Acetaldehyde; GLY: Glycerol; H.ALC: Higher Alcohols; ETH.A: Ethyl acetate; D12 and D16: resistance to 12 and 16% ethanol. Strains circled in yellow were excluded, while the ones circled in blue, green and red were retained for further analysis (for details, check the text hereafter).

We performed a principal component analysis (PCA) according to the oenological parameters in order to achieve a representation of the strains that would facilitate their selection. We used the averages obtained per fermenting parameter and per strain (Tables S5 and S6), and we eliminated those parameters that did not vary between strains (alcoholic degree, total acidity and residual sugars—see Section 3.6). We also took into account the correlations found between the studied parameters to eliminate further criteria from PCA analysis. Indeed, significant positive correlations (significance level = 0.01) were observed between resistance to alcoholic degrees of 12 and 14 on the one hand as well as 14 and 16 on the other hand. Moreover, n-propanol, isobutanol and isoamyl alcohol were all three correlated with the sum of higher alcohols. Consequently, and in addition to the previous three parameters that did not vary between strains, we eliminated from the following analysis resistance to 14% ethanol as it was positively correlated with both 12 and 16% ethanol, and we only considered the sum of higher alcohols, as it was positively correlated with the three higher alcohols measured. PCA representation was subsequently constructed with the remaining 12 candidate strains and 6 parameters (Figure 5).

At low concentrations, higher alcohols (24.38% contribution to axis F1) contribute to the aromatic complexity of wines, whereas high values, usually exceeding 350–400 mg/L (depending on the wine type and the grape cultivar) adversely affect wine bouquet by giving it a grassy taste and heavy solvent odours [63,69]. Strain S15 (red oval in Figure 5) produced the highest value compared to other strains (significant difference at *p* = 0.05) reaching 301.26 mg/L.

Acetaldehyde (33.41% contribution to axis F2) is a product of alcoholic fermentation and it represents 90% of total wine aldehydes. Its low concentrations in wine can contribute to a fruity aroma and to the intensity and colour stability of wine by enhancing (in association with pyruvic acid) the production of the highly stable pigments, vitisins. It may, however, at higher concentrations usually exceeding 100–125 mg/L, lead to the occurrence of turbidity as well as sharp, oxidized, herbaceous and rotten apples aromas [63,69]. In addition, acetaldehyde has a strong affinity for sulphur dioxide used as an antimicrobial and antioxidant agent, which may reduce its effectiveness [70]. Strains S4, S13, S14 and S16 (yellow group in Figure 5) produced significantly higher acetaldehyde concentrations (115.24 to 145.08 mg/L) than the other maintained strains in the selection scheme (Table S6) and could be overlooked in favour of less productive strains.

Ethyl acetate (67.85% contribution to axis F3) is the main ester found in wines. It is responsible of an acetic flavour that turns into a solvent flavour at high values approaching 150–200 mg/L. Concentrations ranging from 50 to 80 mg/L can nonetheless be desirable for wine aroma [63,71,72]. The values that our strains produced of ethyl acetate (between ~20 and 45 mg/L) did not reach high detrimental values.

Glycerol (57.26% contribution to axis F2) is the most abundant compound obtained by fermentation, after ethanol and CO_2_. It plays an important role in wine structure as it gives body and roundness while alleviating harshness and astringency. It is generally produced at concentrations between 5 and 8 g/L [63,71,73]. Our strains fall within these values, the lowest being of 4.75 g/L (S17) (excluding the two strains, S1 and S11, that did not finish the fermentations) and the highest (S14) being close to 8 g/L.

Ethanol is one of the most important factors responsible for fermentations arrest. A decrease in yeast viability due to ethanol is also accompanied by a decrease in fermentation capacity. It has been suggested that the sequential growth of various strains in natural fermentations may be influenced by increasing ethanol concentrations, and the most resistant strains may be those who dominate at the end of fermentations. It has therefore been proposed that this behaviour be taken into account in the creation of mixed cultures for wines inoculation [74]. Two strains (S15 and S20) stand out from the rest as they have the highest viabilities at 16% ethanol and among the highest viabilities at 12% (Table S5)

### 3.8. Proposition of Strains for Selection

The remaining eight strains (522D and seven indigenous strains: S2, S3, S12, S15, S17, S18, S20—blue, green and red ovals in Figure 5) did not have a prejudicial character sufficient to rule them out according to the criteria we adopted. Six out of the seven Lebanese strains (blue oval in Figure 5) did not show significant differences between them and strain 522D for the majority of parameters.

As for strain S15, it was found in extreme positions for many parameters (red circle in Figure 5). It produced the highest concentrations of ethyl acetate and higher alcohols (at the limit of prejudicial concentrations), as well as an experimental wine rich in glycerol (among the highest concentrations). On the other hand, it was characterized by an average production of acetaldehyde (50 mg/L). These parameters could make it a good candidate for selection unless the higher alcohol concentration it produced adversely affect wine aroma. To verify this, the experimental wine of strain S15 was tasted and compared to that of strain S20 (green circle in Figure 5) found in the 522D strain group for all parameters except viabilities at 14 and 16% ethanol. While S15 and S20 did not show significant differences in acetaldehyde and glycerol productions, they significantly differed by the sum of higher alcohols and ethyl acetate (S20 producing one of the lowest higher alcohol concentrations and the lowest ethyl acetate concentration, while S15 having the highest production for both). Finally, S15 and S20 had respectively the highest and the second highest viabilities of the eight strains at 14 and 16% ethanol.

The experimental wines of these two strains were therefore subjected to a paired sensory comparison test which revealed that both strains produced wines that had no undesirable flavours and that did not differ in tannins or body. However, strain S15 wine’s aroma was more expressive than that of strain S20, which was more discreet. This might be partly linked to their productions of higher alcohols and ethyl acetate. Since these two strains did not present detrimental features according to the technological, aromatic and sensory parameters we adopted, they can be further tested, particularly in pilot-scale assays and additional sensory analysis to confirm their adequacy. The subsequent choice of one of the strains would be based on the desired application.

## 4. Discussion

Numerous studies have looked into different aspects of *Saccharomyces cerevisiae*’s genetic diversity since the first application of molecular tools, some three decades ago, which made it possible to conduct such explorations, the scale of which grew over time. Many of these studies focusing on oenological *S. cerevisiae* communities have looked into vineyard floras [17,33,35,43,75,76,77,78,79] collected to conduct fermentations in the lab, and only a few have explored the fermenting floras in their natural environment, i.e., the wineries [21,22,23], while some have looked at both populations [19,27,41,80]. Even if a constant circulation and exchange of strains is most probable between wineries and vineyards [19,27,43], wineries might also have their own implanted yeast lineages that conduct or participate in the natural fermentations and are different from the surviving ones in the vineyards [27,34,80,81,82].

In this study, we chose to look at the *S. cerevisiae* fermenting flora responsible for the natural fermentations conducted in their local wineries, regardless of the origin of their introduction, whether it was the vineyard or the winery. We first assessed the genetic diversity over the whole Lebanese territory, encompassing all regions still producing naturally fermented wines with no starter addition, an ancestral but still ongoing practice. A pronounced biodiversity was revealed within *S. cerevisiae* fermentative population dispatched across the territory on the basis of both interdelta and microsatellites analysis. Diversity figures from similar surveys concerning wineries in other oenological regions are different from one study to the other (some of the variability factors probably being the use of different methods and different sampling schemes), but all such reports agree on high *S. cerevisiae* diversities [20,21,22,23,55,83].

The diversity of the oenological flora that we observed over the Lebanese territory reflects a diversity that exists within single fermentations, since various interdelta patterns were encountered in the same fermenting vats with a predominance of few patterns in most of the vats analysed. These observations are consistent with the results of several reports [22,25,27,28,29,30,84], although the occurrence of dominant isolates is not systematic, as pointed out by other authors [29,32] and as we saw in one of two years in winery HY. Population equilibrium thus seems to be variable from one alcoholic fermentation and one year to the other. It further appeared that isolates circulate within wineries and contaminate different vats, which seems to occur easily (probably via insects, equipment, workers, etc.), even if care is taken to prevent such cross-contamination [25,29]. Some of the strains also reappeared in a number of the studied wineries over multiple years, whether consecutive or not, but dominant strains did not necessarily seem more prone to surviving and colonizing the next-year fermentations, as it has been previously proposed [22], since perennial strains were not always dominant in our study. The occurrence of resident strains associated with wineries is not a universally observed phenomenon, but it has already been described elsewhere [16,17,23,29,30,32,85]. It could be explained by the presence of a resident flora over at least the winery equipment, as it has been previously proposed [29,30,85], and as it was recently observed by Abdo et al. [86], who found a pre-existing *S. cerevisiae* flora on second-hand vinification equipment recovered for use in a newly established winery and which may have subsequently served as a microbial reservoir.

Our findings of dominance, vats cross-contaminations and perenniality of strains, along with the observation of winery-linked patterns and lineages, support the idea of winery related communities conducting the fermentations that we refer to as a “winery effect” on strain relatedness. The term winery points here to the smallest entity we studied and not to the source of the lineages, which might originate from the winery itself or the vineyard, a question we did not address.

At the larger scale of vicinal wineries, some isolates were found to be related, and common strains could be found between wineries in a 5- to 25-km range. This could reflect spontaneous contamination from one location to the other by insects, such as wasps or honey bees, that could disseminate *S. cerevisiae* strains up to a distance of approximately 10 km [87,88]. The presence of common strains over larger distances could be associated with birds or human-related activities [87,89] that could transport them farther than insects. The scattering of related strains over a limited geographical area was accompanied by the loss of observable strains relatedness over a larger range, as it was observed in the Charentes area [22]. Our detection of identical or sibling isolates over small geographical areas is also in line with the observations of Torija et al. [23], who detected identical strains in different cellars of areas within a 50-km range. Moreover, Schuller et al. [35] found that strains of *S. cerevisiae* originating from vineyards in close proximity (5–10 km) tended to be less divergent than strains from remote locations, while Börlin et al. [19] observed a smaller differentiation between *S. cerevisiae* populations of two adjacent wine estates in comparison to the population of a more distant site.

Neither interdelta nor microsatellites analysis established any link between wine type and strain relatedness, suggesting that the fermenting Lebanese oenological local flora was interrelated regardless of the wine process. On the other hand, and according to interdelta analysis, non-oenological strains seemed more related to each other than to Lebanese isolates. This is in agreement with previous conclusions on the clustering of *S. cerevisiae* strains according to the thriving media [5,7,90,91], which exert specific selective pressures, leading to a differential evolution of the lineages involved. In this regard, oenological floras remain interrelated regardless of the country of origin, even though a geographic differentiation may exist within this community [5].

When considering the Lebanese flora in comparison to oenological worldwide floras, microsatellites analysis suggested that a specific Lebanese group might be distinguished from non-Lebanese oenological strains. Even if a limited clustering was occasionally observed in other regions or countries with comparable populations, the clustering of Lebanese strains was wider in terms of frequency, isolate numbers, and geography. The Lebanese flora had previously been shown to be ancestral to other oenological floras [5], and probably at their origin, in accordance with the “Mesopotamian” hypothesis of wine strain emergence, as well as their subsequent dispersion through grapevine migration [5,7,8]. The specific clustering of the Lebanese flora may be the result of a rather confined evolution away from an extensive exchange of genetic material with other populations, from the time of its first involvement in winemaking. This confined evolution may itself be linked, among other factors, to the geographic distance between the Lebanese flora and the other communities analysed in this study, as well as to the still-limited use of commercial oenological starters at the time of the study, and their concentration to a main single region of industrial wineries, which we did not take samples from. It could also be related to intermixing within the Lebanese territory, which homogenizes the yeast population, since exchange of grapes to be used in natural fermentations between different geographical areas is common. Indeed, musts used for winemaking may originate from the grapes adjacent to the winery itself or from grapes bought from other Lebanese regions. This might also explain the loss of relation with geography within the Lebanese territory and the reason why the main observed link between strains relatedness was the “winery effect”.

In parallel to the analysis of genetic diversity, we assessed oenological features of a few Lebanese strains. Genetic differentiation has been shown to be accompanied by a phenotypic differentiation [45,46], which justifies the actual trend suggesting the use of indigenous strains for improving the sensory characteristics of wines by the production of typical and distinguished aromas linked to specific regions or wine types [48,49,50,51,52]. From this perspective, we tested the potential of 21 representative indigenous strains to be used as starters for winemaking according to a pre-selection scheme assessing technological and organoleptic characteristics during lab-scale fermentations.

Our results showed that the Lebanese indigenous strains of *S. cerevisiae* differed with respect to their technological and aromatic characteristics. The observation of such feature differences is in line with many previous studies that have explored various commercial or indigenous *S. cerevisiae* strains. The values of the different aromatic molecules detected in our experimental wines were within the ranges usually reported in the literature [48,49,92,93,94]. The tests we carried out allowed us to eliminate 14 strains that could have undesirable effects on the progress of the fermentations or on the organoleptic quality of the wines produced on our musts. Seven indigenous strains passed our pre-selection scheme, two of which had their experimental wines tasted. One of them in particular, strain S15, exhibited interesting aromatic characteristics, produced expressive experimental wine, and showed good resistance to high alcoholic degrees. It may therefore have a good potential for general winemaking, or for particular applications such as the production of sweet wines with high alcoholic degrees, a practice still common in Lebanon. It should thus be further tested and submitted to pilot-scale assays to confirm industrial use adequacy. It would also be relevant to carry out tests on various grape varieties, local ones in particular, to evaluate the possible contribution of Lebanese strains to the typicality of wines produced in Lebanon.

## Figures and Tables

**Table 1:** Various diversity aspects studied within the *Saccharomyces cerevisiae* Lebanese flora, with interdelta fingerprinting.

Inter-Diversity Aspect	Number of Years	Number of Villages	Number of Wineries	Number of Vats per Winery and per Year	Number of Isolates per Winery and per Year	Winery Name	Year Considered
Biogeographical diversity survey	4	51	79	1 to 6	1 to 14	See Figure S1	Years I, II, III, IV
Diversity within wineries ‡	2	9	9 *	1	9 or 10	Winery M	Years III and IV
						Winery MC	Year IV
						Winery WKa	Year IV
						Winery A	Year IV
						Winery T	Year IV
						Winery B	Years III and IV
						Winery HY	Years III and IV
						Winery DG	Year III
						Winery D	Year III
Perenniality in wineries ‡	2 to 4	7	7 *	1 to 6	4 to 14 **	Winery M	Years I, II, III, IV
						Winery MC	Years I, III, IV
						Winery WKa	Years III and IV
						Winery A	Years III and IV
						Winery T	Years III and IV
						Winery B	Years III and IV
						Winery HY	Years III and IV

‡ Table S1 provides extensive details about the second and third aspect, * One winery per village and per year, ** Numbers below 4 were not considered during analysis, even if available.

## Supplementary Materials

The following are available online at https://www.mdpi.com/article/10.3390/jof7080678/s1, Figure S1: Sampling spots over the Lebanese territory, Table S1: Diversity according to interdelta analysis in 10 wineries over several years, Table S2: Non-Lebanese strains included in interdelta analysis, Table S3: Origins of non-Lebanese strains according to country and production site for microsatellites analysis, Table S4: Strains used in lab-scale fermentations, Table S5: Technological characteristics of the 22 studied *S. cerevisiae* strains and basic features of wines, Table S6: Aromatic compounds (mg/L) in experimental wines produced by the 22 *S. cerevisiae* strains during lab-scale fermentations.
