# Supplementary material for: Lebanon’s Native Oenological Saccharomyces cerevisiae Flora: Assessment of Different Aspects of Genetic Diversity and Evaluation of Winemaking Potential
Source: J Fungi (Basel). 2021 Aug 23;7(8):678. doi: 10.3390/jof7080678 (PMC8398109; doi:10.3390/jof7080678)
Supplement: Supplementary file 1 [file jof-07-00678-s001.zip › jof-1279881-supplementary.pdf]

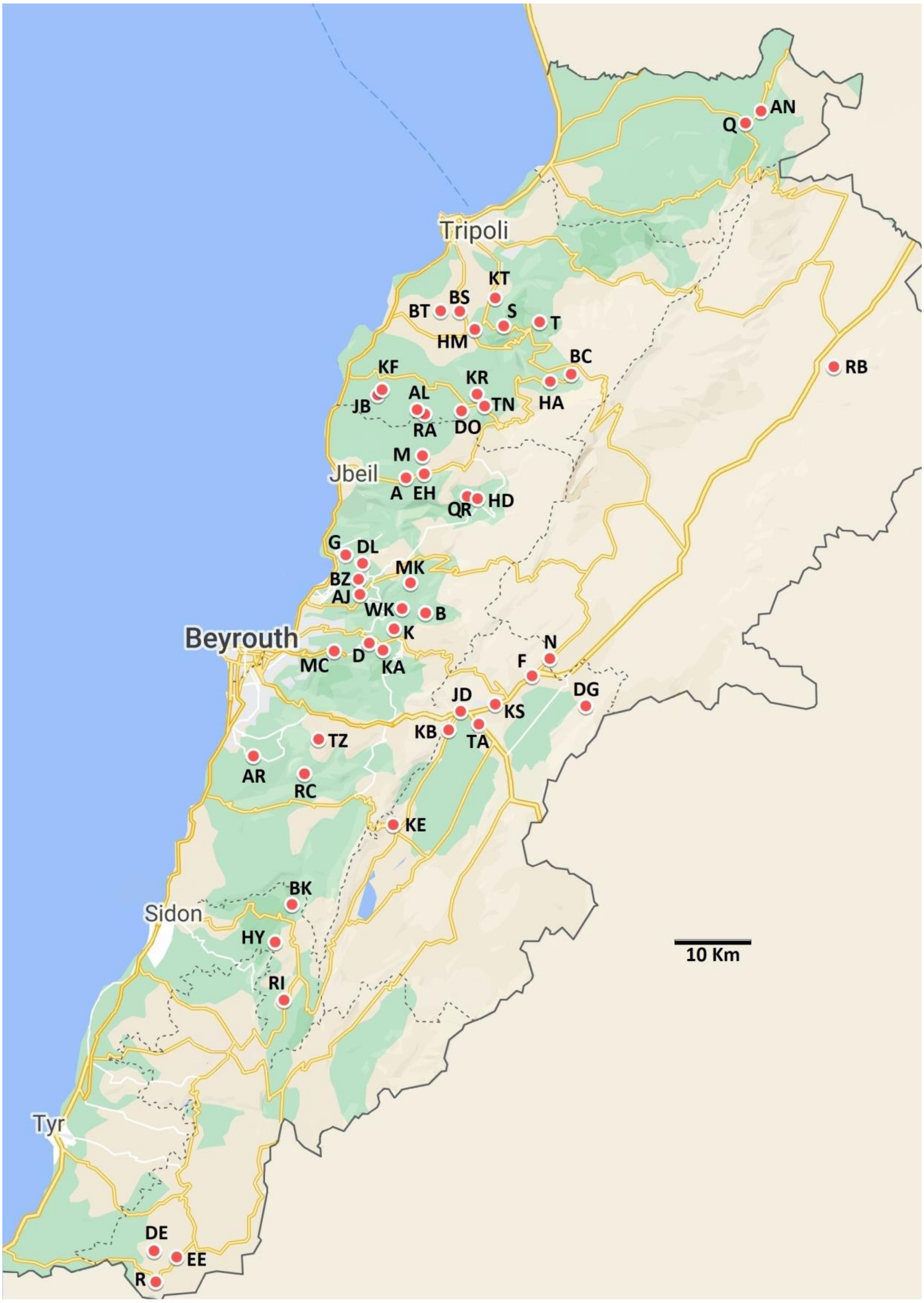

Figure S1 Sampling spots over the Lebanese territory

Villages from where the samples were collected are indicated on the map by their initials

Table S1 Diversity according to Interdeltas analyses in 10 wineries over several years

	Year IV				Year III				Year II				Year I			
	**Vat**	**Nb.I**[1]	**ID.P**[2]	**%** [3a]	**Vat**	**Nb.I**	**ID.P**	**%** [3b]	**Vat**	**Nb.I**	**ID.P**	**%**	**Vat**	**Nb.I**	**ID.P**	**%**
**Winery M**	*a*	10	**PM1**	40%	*a*	10	**PM8**	20%	*a*	2	**PM17**	50%	*a*	1	PM20	25%
			PM2	10%			PM9	10%			PM18	25%	*b*	1	PM8	25%
			PM3	10%			PM10	10%	*b*	2	**PM17**	50%	*c*	2	PM21	25%
			PM4	10%			PM11	10%			PM19	25%			PM22	25%
			PM5	10%			PM12	10%								
			PM6	10%			PM13	10%								
			PM7	10%			PM14	10%								
							PM15	10%								
							PM16	10%								
**Winery MC**	*a*	10	**PMC1***	100%	*a*	2	**PMC1**	57%	*a*	1	**PMC1** ‡		*a*	1	**PMC4**	50%
					b	3	**PMC2**	28%					*b*	2	**PMC4**	50%
							PMC3	14%							PMC5	25%
					c	2	**PMC1**	57%					*c*	1	PMC6	25%
**Winery WKa**	*a*	10	**PWKa1**	80%	*a*	4	PWKa1	75%	*a*	1	PWKa5‡		*a*	1	PWKa6‡	
			PWKa2	10%			PWKa4	25%								
			PWKa3	10%												
**Winery A**	*a*	10	**PA1**	20%	*a*	2	PA3	7%	*a*	1	PA17‡					
			**PA2**	20%			PA9	7%								
			PA3	10%	*b*	2	**PA4**	14%								
			PA4	10%			PA10	7%								
			PA5	10%	*c*	4	**PA4**	14%								
			PA6	10%			PA5	7%								
			PA7	10%			**PA11**	14%								
			PA8	10%			PA12	7%								
					d	2	PA6	7%								
							PA13	7%								
					e	2	**PA11**	14%								
							PA14	7%								
					f	2	PA15	7%								
							PA16	7%								
**Winery T**	*a*	10	**PT1**	30%	*a*	2	PT7	16%	*a*	1	PT12‡	100%				
			**PT2**	30%			PT8	16%	*b*	1	PT12‡	100%				
			PT3	10%	*b*	4	**PT9**	33%								
			PT4	10%			PT10	16%								
			PT5	10%			PT11	16%								
			PT6	10%												

**Winery B**	*a*	10	**PB1**	50%	*a*	10	**PB5**	30%		
			**PB2**	30%			**PB6**	20%		
			PB3	10%			PB7	10%		
			PB4	10%			PB8	10%		
							PB9	10%		
							PB10	10%		
							PB11	10%		
**Winery HY**	*a*	10	PHY1	10%	*a*	10	**PHY11**	20%		
			PHY2	10%			PHY1	10%		
			PHY3	10%			PHY2	10%		
			PHY4	10%			PHY12	10%		
			PHY5	10%			PHY13	10%		
			PHY6	10%			PHY14	10%		
			PHY7	10%			PHY15	10%		
			PHY8	10%			PHY16	10%		
			PHY9	10%			PHY17	10%		
			PHY10	10%						
**Winery DG**					*a*	9	**PDG1**	33%		
							**PDG2**	22%		
							PDG3	11%		
							PDG4	11%		
							PDG5	11%		
							PDG6	11%		
**Winery D**					*a*	9	**PD1**	44%		
							PD2	11%		
							PD3	11%		
							PD4	11%		
							PD5	11%		
							PD6	11%		

1- **Nb.I :** Number of studied isolates

2- **ID.P :** Interdelta Profile (isolates are referenced by their profiles pattern and not their code)

3- **% :** Profile proportion in the sample when only one is considered per winery (3a), or in the population of all samples when several are included from the same winery (3b)

* Profiles in bold are dominant in the populations of the studied wineries (from the same vat or from several vats of the same winery) while underlined profiles are stable for more than a year in the same winery

‡ Not taken into account for dominance or perenniality observation

Table S2 Non-Lebanese strains included in Interdelta analysis

	Source/Reference	Place of Isolation
**Non-oenological strains**		
CLIB 409	Sugar cane	Vietnam
CLIB 412	Sake	Japan
CLIB 413	Fermented rice	China
CLIB 414	High-sugar food	Japan
CLIB 415	Fermented rice	Taiwan
**Oenological strains***		
Levuline BRG	UP3OY5	Bourgogne, France
Levuline CER	Isolate of Davis 522	University of California, Davis
Levuline CHP	CIVC8130	Champagne, France
Ceres C2C	-	Languedoc / INRA Narbonne
Levuline C19	C19	INRA Colmar - INRA Montpellier
EG8	EG8	Alsace / INRA Colmar
Levuline FB	GLO 806	Champagne, France
Levuline Killer	GLO 522 K	-
Montbazin 1	-	-
Levuline Primeur	GLO 7447	-
522 Davis	522 Davis	University of California, Davis
**Other strains**		
S288C	Plant	USA
CBS 1171NT	Beer	Netherlands
CBS 1907	-	-

Table S3 Origins of non-Lebanese strains according to country and production site for microsatellites analysis

**Oenological isolates**			**Number of strains**											
**Country**	**Region/Village**	**Total**	W1*	W2	W3	W4	W5	W6	W7	W8	W9	W10	W11	W12
Italy		**18**	18											
South Africa	Cap	**18**	2	2	3	2	4	5						
Spain		**28**												
	Priorat	9	9											
	Terra Alta	9	9											
	Alicante	4	4											
	Pontevedra	1	1											
	Galicia	1	1											
	Huelva	1	1											
	Manzanares	1	1											
	Valladolid	1	1											
	Jumilla	1	1											
USA		**15**	1	4	1	1	1	6	1					
France		**158**												
	Alsace	90	14	14	5	9	2	14	12	7	13			
	Champagne	2	2											
	Cognac	7	2	1	1	1	1	1						
	Montpellier	19	19											
	Nantes	18	1	2	1	2	1	1	2	1	2	1	2	2
	Côtes du Rhone	22	14**	4**	1	1	1	1						

* Winery 1, 2 etc…
** 2 years

Table S4 Strains used in lab-scale fermentations

Strain	Winery	Wine type	Isolation year
**S1**	BCa	White	III
**S2**	BCb	White	III
**S3**	BCc	White	III
**S4**	DL	White	III
**S5**	EH	White	III
**S6**	HAa	White	III
**S7**	JDc	White	I
**S8**	JDa	Rosé	III
**S9**	JDa	Red	III
**S10**	Ka	Sweet	III
**S11**	Ka	Sweet	III
**S12**	Kb	White	III
**S13**	KE	White	III
**S14**	MC	Red	III
**S15**	MC	Sweet	I
**S16**	MC	Red	I
**S17**	R	Rosé	III
**S18**	Ta	Sweet	III
**S19**	Ta	Sweet	III
**S20**	WKa	Red	I
**S21**	WKb	Red	III

Table S5 Technological characteristics of the 22 studied *S.cerevisiae* strains and basic features of experimental wines.

Strain	Glycerol		AD*		RS**		T.A***		R12⁑		R14⁑		R16⁑		H_2S‡
**S1**	4.64	±0.01	9.23	±0.06	35.65	±2.45	8.87	±0.12	ND		ND		ND		L
**S2**	6.34^{ab}	±0.57	11.08	±0.14	2.25	±0.85	8.62	±0.13	72.92^{bc}	±10.14	62.50	±7.30	0.00^{a}	±0.00	L
**S3**	6.64^{ab}	±0.14	10.83	±0.10	2.25	±0.55	8.75	±0.12	21.78^{a}	±5.61	20.00	±2.37	17.78^{ab}	±1.42	L
**S4**	6.73^{ab}	±0.09	10.86	±0.01	1.95	±0.25	8.98	±0.09	64.19^{bc}	±8.77	54.74	±7.28	14.32^{ab}	±5.00	L
**S5**	6.62	±0.35	10.92	±0.06	2.40	±1.00	8.75	±0.24	58.33	±9.13	53.33	±5.62	51.67	±8.40	M
**S6**	6.30	±0.14	10.84	±0.18	1.20	±0.60	7.87	±0.00	29.74	±4.36	20.43	±2.64	0.00	±0.00	M
**S7**	6.58	±0.01	10.99	±0.04	2.15	±0.45	8.62	±0.00	75.00	±8.94	43.75	±2.24	0.00	±0.00	M
**S8**	6.87	±0.20	11.06	±0.10	1.10	±0.10	8.69	±0.43	63.64	±3.28	42.42	±5.29	30.30	±2.20	H
**S9**	6.73	±0.24	10.91	±0.06	2.90	±1.00	8.75	±0.26	65.00	±7.44	65.00	±6.88	37.50	±4.35	M
**S10**	7.83	±0.20	10.79	±0.10	2.10	±0.30	9.56	±0.19	36.73	±6.52	36.36	±3.33	27.27	±4.13	H
**S11**	4.53	±0.24	7.86	±0.16	53.25	±1.05	8.31	±0.31	ND		ND		ND		L
**S12**	6.47^{ab}	±0.42	10.96	±0.09	2.65	±1.35	8.44	±0.18	71.88^{bc}	±9.47	34.38	±5.12	32.29^{bc}	±4.56	L
**S13**	6.34^{ab}	±0.18	11.00	±0.11	2.54	±0.16	9.01	±0.21	32.33^{ab}	±9.52	15.86	±1.92	12.51^{ab}	±1.49	L
**S14**	7.56^{b}	±0.42	10.91	±0.12	1.70	±0.10	9.12	±0.12	9.07^{a}	±3.46	6.67	±2.04	6.00^{a}	±2.11	L
**S15**	7.16^{b}	±0.04	10.97	±0.15	1.65	±0.75	8.75	±0.13	76.71^{c}	±8.23	75.34	±5.67	72.60^{d}	±6.97	L
**S16**	6.62^{ab}	±0.25	10.87	±0.11	2.10	±0.20	9.06	±0.15	34.88^{abc}	±4.71	16.28	±3.46	0.00^{a}	±0.00	L
**S17**	4.75^{a}	±0.77	10.91	±0.14	4.65	±0.65	8.56	±0.44	40.00^{abc}	±8.23	40.13	±6.31	32.00^{bc}	±6.34	L
**S18**	5.67^{ab}	±0.05	11.17	±0.14	2.35	±0.55	8.94	±0.19	73.75^{bc}	±7.56	50.00	±5.45	4.63^{a}	±1.85	L
**S19**	5.87	±0.10	11.04	±0.17	2.20	±0.20	9.12	±0.24	67.14	±6.82	55.71	±7.26	0.00	±0.00	M
**S20**	7.23^{b}	±0.18	11.08	±0.15	2.40	±0.40	8.28	±0.40	72.04^{bc}	±5.76	67.59	±7.02	51.60^{c}	±4.38	L
**S21**	7.55	±0.59	10.97	±0.15	1.55	±0.55	8.50	±0.25	27.42	±5.96	17.74	±4.01	0.00	±0.00	M
**522D**	5.72^{ab}	±0.30	10.67	±0.18	9.00	±2.30	8.94	±0.81	41.18^{abc}	±5.1	23.53	±2.93	0.00^{a}	±0.00	L
**Pr > F**	<0.0001		<0.0001		<0.0001		0.157		<0.0001		<0.0001		<0.0001		
**Significant §**	Yes		Yes		Yes		No		Yes		Yes		Yes		

Data are means ± standard deviation of two independent experiments

§ Given a statistical significance level of 5% and the p-value of the F statistic in one way ANOVA analysis

For the parameters and strains retained for PCA analysis, different superscript letters along a column indicate significant differences between strains according to the Tukey test.

* Alcohol Degree in % (v/v)

** Residual sugars in g/l

*** Total Acidity in g/l

⁑ Resistance to 12 (R12), 14(R14) and 16(R16) % ethanol

‡ H_2S production : L, M, H stand respectively for low, medium and high productions of H_2S (see Materials and Methods)

ND Not Determined

Table S6 Aromatic compounds (mg/l) in experimental wines produced by the 22 studied *S.cer*evisiae strains during lab-scale fermentations.

Strain	n-Propanol		Isobutanol		Isoamyl Alcohol		Sum of Higher Alcohols		Ethyl Acetate		Acetaldehyde	
**S1**	46.32	±1.46	54.67	±1.68	174.65	±8.94	275.64	±12.08	43.38	±0.33	38.17	±1.97
**S2**	34.35	±2.26	52.23	±3.32	162.68	±18.67	249.26 [bc]	±24.24	29.51 [abcd]	±2.08	36.37 [a]	±1.49
**S3**	34.66	±2.71	58.04	±1.47	113.29	±6.38	205.99 [ab]	±10.57	30.73 [abcd]	±0.57	37.39 [a]	±3.23
**S4**	39.92	±0.51	24.16	±0.46	149.98	±11.47	214.06 [abc]	±10.50	20.55 [ab]	±1.38	115.24 [c]	±2.72
**S5**	44.45	±5.17	46.59	±1.71	152.12	±11.48	243.17	±4.60	31.65	±2.24	52.74	±3.73
**S6**	28.10	±1.44	51.78	±1.77	120.54	±3.68	200.42	±0.47	27.98	±2.48	39.20	±1.00
**S7**	40.80	±3.19	14.39	±1.64	124.69	±14.85	179.88	±10.02	22.93	±2.16	149.12	±5.40
**S8**	39.59	±1.44	48.44	±2.02	155.24	±1.22	243.28	±2.24	34.35	±2.66	53.45	±1.79
**S9**	52.91	±4.40	45.56	±3.95	124.63	±4.79	223.10	±3.56	21.59	±1.73	65.08	±4.66
**S10**	46.43	±0.96	75.42	±2.60	141.25	±15.27	263.10	±11.72	24.95	±3.92	88.03	±4.41
**S11**	36.60	±0.78	44.68	±0.42	131.99	±6.17	213.26	±6.53	27.72	±1.58	34.92	±4.80
**S12**	46.59	±5.50	49.04	±6.84	148.18	±14.70	243.81 [abc]	±27.03	34.28 [cde]	±4.27	38.46 [a]	±0.46
**S13**	32.67	±0.50	27.29	±3.00	192.57	±16.91	252.53 [bc]	±19.41	20.31 [ab]	±1.02	145.08 [c]	±15.29
**S14**	38.09	±1.18	43.09	±2.70	130.49	±0.51	211.67 [abc]	±3.38	20.61 [ab]	±0.18	122.52 [c]	±4.60
**S15**	63.84	±3.52	90.61	±3.58	146.82	±4.17	301.26 [c]	±11.26	45.24 [e]	±1.77	41.14 [ab]	±2.99
**S16**	27.21	±3.45	53.60	±4.08	101.92	±4.36	182.73 [ab]	±11.89	40.11 [de]	±3.01	127.43 [c]	±4.24
**S17**	38.00	±3.13	29.08	±2.55	136.71	±13.88	203.79 [ab]	±14.46	24.05 [abc]	±2.29	47.25 [ab]	±4.51
**S18**	45.36	±4.13	43.47	±6.35	126.47	±12.59	215.30 [abc]	±23.06	31.17 [bcd]	±3.13	61.45 [ab]	±4.94
**S19**	29.37	±2.29	42.58	±2.44	146.59	±5.06	218.54	±0.32	28.17	±1.01	36.27	±0.54
**S20**	39.16	±1.20	35.78	±0.96	125.09	±5.23	200.03 [ab]	±4.99	19.03 [a]	±0.71	63.83 [ab]	±6.20
**S21**	28.55	±1.92	40.84	±3.31	163.22	±8.07	232.61	±13.30	29.27	±0.64	40.34	±1.96
**522D**	28.28	±1.75	25.22	±1.81	101.81	±14.78	155.30 [a]	±18.33	23.34 [abc]	±1.48	74.20 [b]	±9.30
**Pr > F**	<0.0001		<0.0001		0.000		<0.0001		<0.0001		<0.0001	
**Significant §**	Yes		Yes		Yes		Yes		Yes		Yes	

Data are means ± standard deviation of two independent experiments

§ Given a statistical significance level of 5% and the p-value of the F statistic in one way ANOVA analysis

For the parameters and strains retained for PCA analysis, different superscript letters along a column indicate significant differences between strains according to the Tukey test.
